# A Handy Method of Sterilising Instruments

**Published:** 1902-12-06

**Authors:** 


### A HANDY METHOD OF STERILISING INSTRUMENTS.

The fact that in heat, applied by boiling or steaming, we have an absolutely trustworthy means of sterilising surgical instruments renders the search for other methods a matter of comparatively small interest to those who work in hospitals. Still there are occasions in which the full resources of the operating theatre are not available, and it becomes a matter of great importance, especially to those practising in the country, to obtain a reliable sterilisation by some less cumbersome means than by boiling, and under such circumstances it will be useful to recall a simple proceeding recently described by Dr. Karl Gerson in the *Deutsche Medicinische Wochenschrift.* The plan which has for a long time been adopted by Professor Mikulicz for sterilising the hands before the performance of surgical operations, namely, by friction with spirit of soap, is now pretty well known, although we believe it is but little employed in this country. As the result of a series of experiments with pus-producing organisms, Dr. Gerson has recently proved the efficacy of spirit of soap as an antiseptic, and has shown that by rubbing instruments with cotton wool moistened with this solution, they can be efficiently sterilised even though known to be infected with staphylococcus aureus and albus, and that by wrapping them up in cotton wool moistened with it they can be kept in an aseptic condition for a considerable time. When the spirit of soap is so applied, the spirit evaporates, leaving the instruments coated, and the wool charged with a dense layer of soap. It is even said that for bougies, catheters and other large instruments, to wrap which in cotton wool might be difficult, rubbing with spirits of soap for three minutes before an operation is sufficient—a statement, by the bye, which to our mind throws doubt upon the whole of Dr. Gerson's conclusions. The septicity of a foul catheter lies within, and it must be quite impossible to sterilise such an instrument with certainty by means of any solution, however germicidal it may be. It is here that heat has the pull, penetrating as it does into the joints and corners of the most complicated apparatus. Still, for solid and smooth instruments there is every reason to believe that the application of spirits of soap will be useful. A somewhat lengthy experience has shown that it does very well for the hands, which are much more septic than any well-cleaned instrument, and there seem to be no grounds for doubting that it will do equally well for any solid instrument, to the whole surface of which it can be directly applied. Spirit of soap, it may be added, is merely hard soap dissolved in dilute alcohol. It is essential to remember, however, that for the removal of the coating of soap left upon the instruments, sterilised water or spirits of wine and sterilised lint or other form of "wipe" must be employed, so that after all, even by this method, one does not entirely get rid of sterilisation by heat.

